# Ground-Glass Opacities in the Access Route and Biopsy in Highly Perfused Dependent Areas of the Lungs as Risk Factors for Pulmonary Hemorrhage During CT-Guided Lung Biopsy: A Retrospective Study

**DOI:** 10.3390/tomography11030035

**Published:** 2025-03-14

**Authors:** Michael P. Brönnimann, Leonie Manser, Andreas Christe, Johannes T. Heverhagen, Bernhard Gebauer, Timo A. Auer, Dirk Schnapauff, Federico Collettini, Christophe Schroeder, Patrick Dorn, Tobias Gassenmaier, Lukas Ebner, Adrian T. Huber

**Affiliations:** 1 Department of Diagnostic, Interventional and Paediatric Radiology, Inselspital, Bern University Hospital, University of Bern, Rosenbühlgasse 27, 3010 Bern, Switzerland; leonie.manser@insel.ch (L.M.); andreas.christe@insel.ch (A.C.); johannes.heverhagen@insel.ch (J.T.H.); lukas.ebner@luks.ch (L.E.); adrian.huber@luks.ch (A.T.H.); 2 Department of Radiology, Charité—Universitätsmedizin Augustenburger Platz 1, 13353 Berlin, Germany; bernhard.gebauer@charite.de (B.G.); timo-alexander.auer@charite.de (T.A.A.); dirk.schnapauff@charite.de (D.S.); federico.collettini@charite.de (F.C.); 3 Clinician Scientist Program, Berlin Institute of Health at Charité—Universitätsmedizin Berlin, Augustenburger Platz 1, 13353 Berlin, Germany; 4 Department of Radiology, Centre Hospitalier du Nord, 120 Av. Lucien Salentiny, Ettelbruck, 9080 Luxembourg, Luxembourg; cschroeder@telemedicineclinic.com; 5 Department of Thoracic Surgery, Inselspital, Bern University Hospital, University of Bern, Freiburgstrasse 4, 3010 Bern, Switzerland; patrick.dorn@insel.ch; 6 Department of Radiology and Nuclear Medicine, Cantonal Hospital Lucerne, University of Lucerne, 6000 Lucerne, Switzerland; tobias.gassenmaier@luks.ch

**Keywords:** hemorrhage, biopsy, image-guided biopsy, risk factors, tomography, radiography interventional

## Abstract

Background/Objectives: The risk of hemorrhage during CT-guided lung biopsy has not been systematically studied in cases where ground-glass opacities (GGO) are present in the access route or when biopsies are performed in highly perfused, dependent lung areas. While patient positioning has been studied for pneumothorax prevention, its role in minimizing hemorrhage risk remains unexplored. This study aimed to determine whether GGOs in the access route and biopsies in dependent lung areas are risk factors for pulmonary hemorrhage during CT-guided lung biopsy. Methods: A retrospective analysis was conducted on 115 CT-guided lung biopsies performed at a single center (2020–2023). Patients were categorized based on post-interventional hemorrhage exceeding 2 cm (Grade 2 or higher). We evaluated the presence of GGOs in the access route and biopsy location (dependent vs. non-dependent areas) using chi square, Fisher’s exact, and Mann–Whitney U tests. Univariate and multivariate logistic regression analyses were conducted to evaluate risk factors for pulmonary hemorrhage. Results: Pulmonary hemorrhage beyond 2 cm occurred in 30 of 115 patients (26%). GGOs in the access route were identified in 67% of these cases (*p* < 0.01), and hemorrhage occurred more frequently when biopsies were performed in dependent lung areas (63% vs. 40%, *p* = 0.03). Multivariable analysis showed that GGOs in the access route (OR 5.169, 95% CI 1.889–14.144, *p* = 0.001) and biopsies in dependent areas (OR 4.064, 95% CI 1.477–11.186, *p* < 0.001) independently increased hemorrhage risk. Conclusions: GGOs in the access route and dependent lung area biopsies are independent risk factors for hemorrhage during CT-guided lung biopsy.

## 1. Introduction

Pulmonary hemorrhage, often self-limited but potentially life-threatening, is the second most common complication (after pneumothorax) of CT-guided lung biopsy [1,2,3]. During CT-guided core biopsy, pulmonary hemorrhage occurs in 30–65% [2,4,5,6] of cases and hemoptysis in 4.1% [1]. While small amounts of hemorrhage around the needle path are common [2,7], they are typically clinically insignificant. In contrast, pulmonary hemorrhages exceeding 2 cm around the needle path (Grade 3 or 4) are fortunately rare [2,8,9,10] but can be potentially life-threatening [11]. Further interventions are required in 6.5% of cases, with a mortality rate of less than 1%. Additionally, it should be noted that early bleeding into the target area can obscure the lesion, potentially compromising or even preventing a successful biopsy. Consequently, identifying risk factors, such as a patient’s anticoagulant medication and procedure-related variables during lung biopsy, is crucial [1].

CT-guided biopsy of ground-glass opacities (GGO) has been associated with pulmonary hemorrhage [2] and clinically significant peri-interventional hemoptysis [12]. GGO results from the occupation of alveolar spaces by cells or fluid and the thickening of alveolar walls [13]. GGO may therefore represent hypervascular areas, pre-existing focal perilesional micro-hemorrhage [14], areas with tumor-related venous congestion [15], or inflammation [16]. Another important factor is the physiology of lung perfusion, as dependent areas of the lung are more perfused due to gravitational forces [17,18,19]. Lesions in the lower lobe have been associated with pulmonary hemorrhage [20] and hemoptysis [21].

However, no study has investigated the occurrence of pulmonary hemorrhage during CT-guided lung biopsy in cases with GGO in the access route or when a biopsy is performed in dependent areas of the lung. This is important, as GGO in the access route may be mitigated, and patients may be positioned to conduct the biopsy in a non-dependent area of the lung. Most studies investigating patient positioning so far have been performed to assess the occurrence of pneumothorax [21,22,23,24,25,26,27,28], but not pulmonary hemorrhage, during CT-guided lung biopsy.

Based on those considerations, we hypothesized that CT-guided lung biopsy with GGO in the access way and in dependent areas of the lung may be associated with a higher occurrence of hemorrhage during lung biopsy. The aim of this study was to investigate the influence of ground-glass opacities in the access route and biopsy in highly perfused dependent areas of the lung as a risk factor for pulmonary hemorrhage during CT-guided lung biopsy.

## 2. Materials and Methods

### 2.1. Study Population

**Figure 1:**
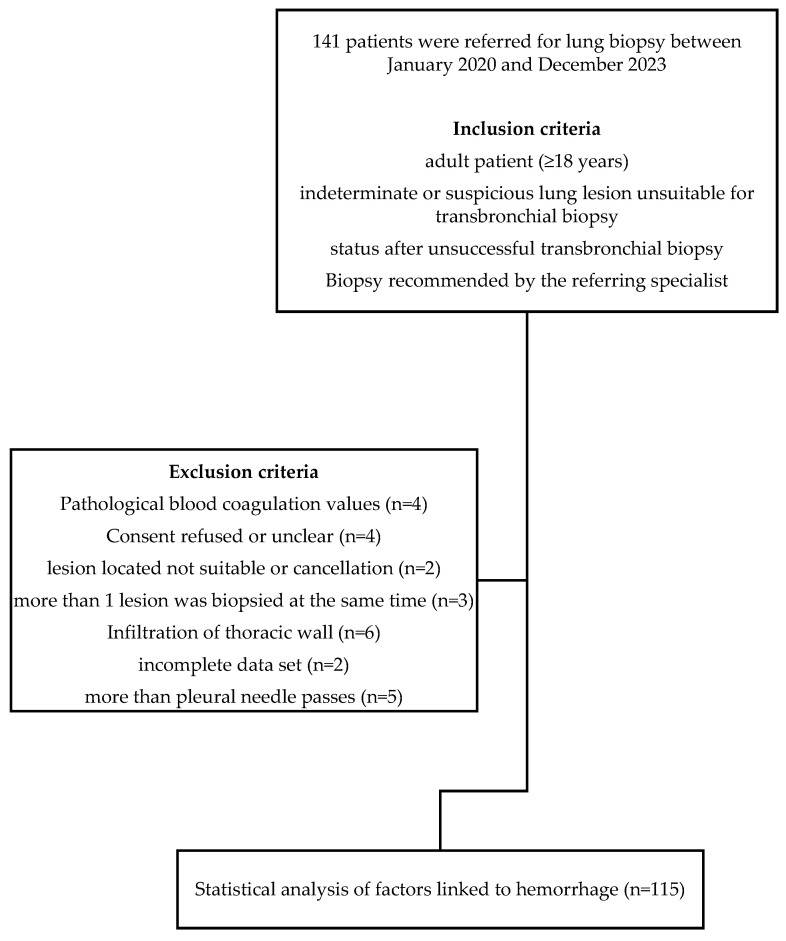
Flowchart showing the study population.

This study retrospectively analyzed 141 percutaneous CT-guided lung biopsies performed at our university hospital between January 2020 and December 2023. Of these, 115 cases met the inclusion criteria, with a mean age of 67 ± 12 years (range 25–89 years; Figure 1).

Among the included participants, 67 were male (58%) and 48 (42%) were female. Sequential exclusion criteria were defined to minimize potential bias caused by significant physiological abnormalities in the pleural cavity or lung parenchyma (Figure 1). In emergency situations or when correction is unsuccessful (e.g., liver cirrhosis, aplasia), lung biopsies are often performed despite unfavorable blood values. Additionally, biopsies with multiple pleural penetrations or those targeting different lesions within the same session, as well as pleural infiltrative processes with potential neovascularization, were excluded due to the elevated risk of initial bleeding.

### 2.2. Initial Assessment

All patients underwent an initial clinical assessment, which included a review of their medical history and standard blood tests. Procedural eligibility criteria required an INR below 1.5 or a Quick value above 60%, a hemoglobin level greater than 80 g/L, and a platelet count exceeding 50 × 10⁹/L, with laboratory values obtained within the last five days. As per clinical guidelines, non-steroidal anti-inflammatory drugs (NSAIDs) and clopidogrel were discontinued five days prior to the procedure, heparin was stopped six hours before, rivaroxaban one day before, and both dabigatran and edoxaban three days before the intervention.

### 2.3. Biopsy Procedure

**Figure 2:**
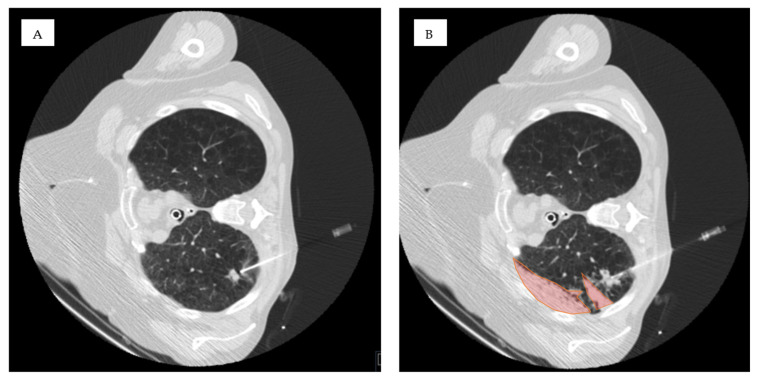
Technical implementation. (**A**) Lesion in the right upper lobe and ipsilateral-dependent patient positioning to prevent a pneumothorax. (**B**) After full core lung biopsy with adjustable penetration depth, a lobar hemorrhage develops (red-colored area).

The biopsies were performed under spontaneous breathing without additional commands as experience has shown that otherwise, compensatory respiratory movements increase, leading to a prolonged procedure time. All procedures were carried out by four experienced interventional radiologists, each with 7 to over 10 years of expertise. CT guidance was used for all biopsies, employing a Toshiba Asteion 4SL scanner. An 18- or 20-gauge semi-automated biopsy system (SemiCut side-cutting; Medical Devices Lease S.A., Zug, Switzerland, or CorVocetTM full-core; Merit Medical Systems, South Jordan, UT, USA) through a matching coaxial needle was used for the interventions. Biopsy planning was based on a non-contrast chest CT with 1 mm reconstruction increments, following standard protocols for optimal needle path selection. Particular attention was given to avoiding pulmonary vessels while crossing pulmonary fissures and targeting areas with pleural effusion was strictly avoided. Patient positioning was determined by the interventionalist, considering both clinical expertise and the patient’s physical condition (Figure 2).

A maximum of 20 mL of 1% lidocaine was used for local anesthesia. To avoid hyperventilation, which could extend the procedure duration, no specific breathing instructions were given. Following tissue extraction, the needle was promptly removed without applying a sealing agent. A post-procedural CT scan was conducted immediately after needle withdrawal, and in the absence of complications, the patient was carefully positioned supine on a bed without further relocation.

### 2.4. Data Collection and Analysis

**Figure 3:**
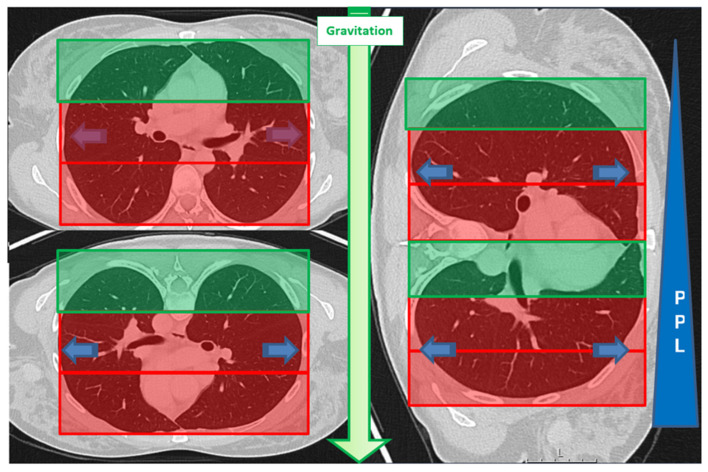
A schematic representation of the zoning approach used in this study based on the position-dependent influence of gravity on pleural pressure (PPL). Pleural pressure increases progressively from the non-dependent upper lung regions to the dependent lower regions. Additionally, gravitational forces contribute to enhanced perfusion in the dependent lung areas [18,19,29]. To simplify classification, only the “RED” zone was designated as dependent. The zoning method followed the rule of thirds.

All procedures were independently reviewed by a board-certified interventional radiologist with eight years of experience and a radiology resident with three years of experience, neither of whom were involved in performing the interventions. Both reviewers were blinded to the patient’s medical histories. A simplified zoning method was applied to differentiate between biopsies in dependent and non-dependent lung regions. The axial planning images of the biopsy, taken with the patient in the final position, were divided into three equal sections using non-anatomical reference points for orientation. The lower two-thirds were classified as dependent lung regions, while the upper third was designated as non-dependent. The section in which the target lesion was located was assessed (Figure 3).

**Figure 4:**
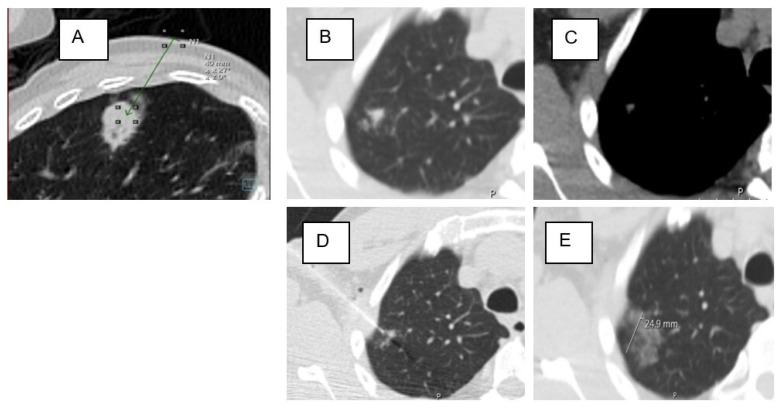
Ground-glass opacification (GGO) in the access route. (**A**) Asymmetric GGO in the access route peripheral to the target lesion. (**B**–**E**) A classic subsolid nodule with surrounding GGO and Grade 2 hemorrhage after biopsy.

**Figure 5:**
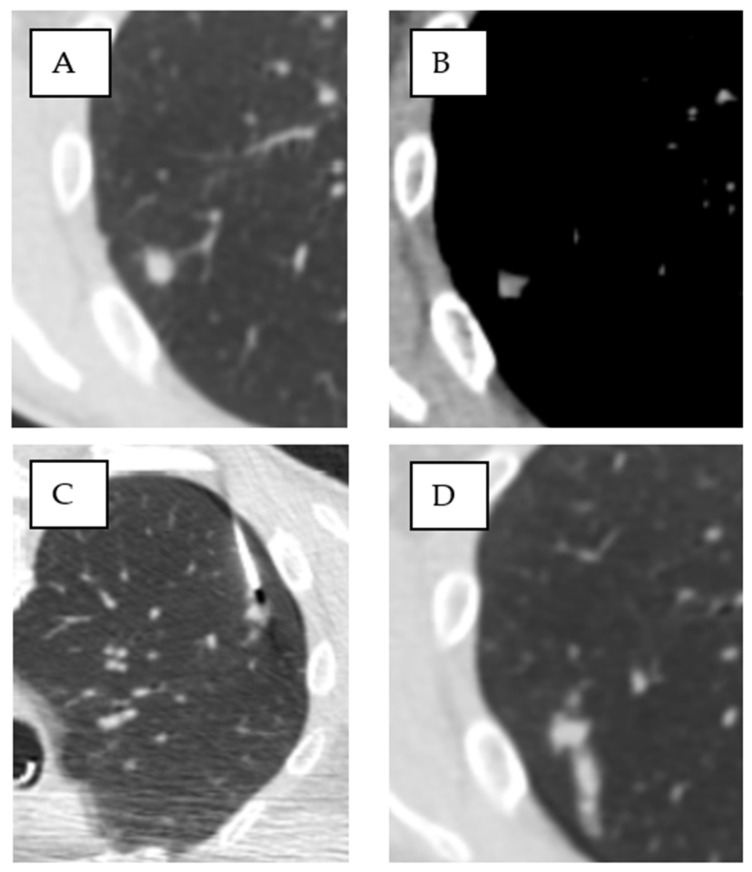
Lesion in the lower lobe without GGO in the access route. (**A**) Small 13 mm suspicious solid lesion in the right lower lobe. (**B**) Target lesion in the corresponding soft tissue window. (**C**) Prone positioning with biopsy. (**D**) Pulmonary hemorrhage Grade 1 in the access route.

The biopsy images, showing the needle within the target lesion, were compared with the planning CT scan. A binary assessment was performed to determine the presence or absence of GGO in the access route. GGO was defined as a hazy increase in lung density without obscuration of the underlying vessels or bronchial walls. If the vessel structures were obscured, the term “consolidation” was applied, and these cases were classified as negative [13,30] (Figure 4 and Figure 5).

**Figure 6:**
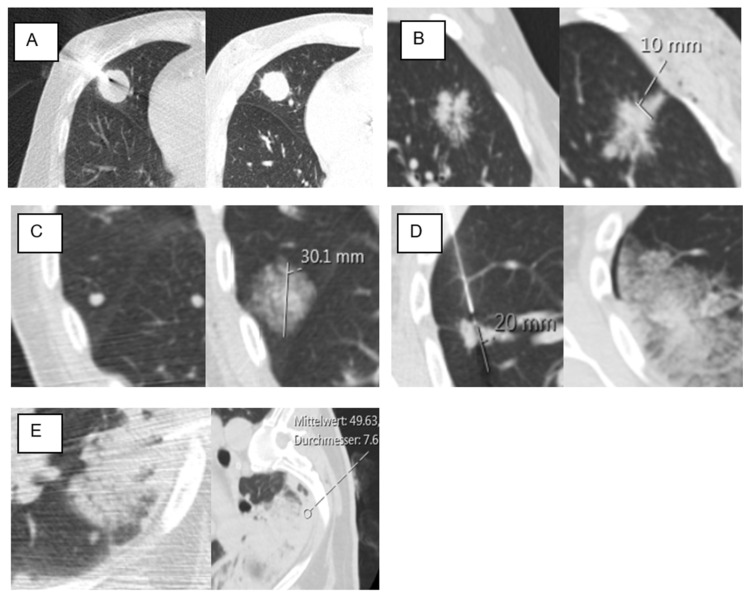
Pulmonary hemorrhage grading system on CT scans performed before/during (**left**) and directly after (**right**) the biopsy pulmonary hemorrhage. (**A**) Grade 0: without image morphological evidence of parenchymal hemorrhage after biopsy. (**B**) Grade 1: new focal hemorrhage less than 2 cm beyond the needle path can be delineated in the access route after the biopsy. (**C**) Grade 2: after the biopsy, the target lesion can no longer be demarcated if the alveolar hemorrhage is locally larger than 2 cm. (**D**) Grade 3: lobar hemorrhage after biopsy. (**E**) Grade 4: hemothorax with immediate hyperdense fluid within the pleural cavity after biopsy [23].

Pulmonary hemorrhage was assessed as new consolidative or ground-glass opacity on postbiopsy images and categorized using a consensus-based grading system adapted from a previous scheme [2,3,5,9]: Grade 0: no pulmonary hemorrhage; Grade 1: needle tract hemorrhage ≤ 2 cm (Figure 6A,B); Grade 2: hemorrhage beyond 2 cm around the needle path, but confined to the sublobar regions (Figure 6C); Grade 3: lobar hemorrhage or larger (Figure 6D); and Grade 4: hemothorax (Figure 6E) [23].

Additional variables evaluated on interventional CT images included the procedure date, patient birthdate, age, lesion size (measured in mm), sex, biopsy angle, distance from the skin to the lesion (mm), distance from the pleura to the lesion (mm), lesion location, and the presence of pulmonary emphysema. Lesions in the middle lobe and lingula were categorized as part of the lower lobe. Information on needle size, biopsy system, and the number of samples was obtained from the intervention report. Histopathological findings from the target lesion, along with the patient’s post-procedural clinical history, were retrospectively retrieved from electronic medical records. Hypervascular lesions included SCLC (small cell lung cancer) and metastasis from primary cancers such as HCC (hepatocellular carcinoma), NET (neuroendocrine tumor)/carcinoid, melanoma, RCC (renal cell carcinoma), breast, thyroid, sarcoma, thymoma, hemangioendothelioma, and germ cell tumor [31,32,33,34,35].

### 2.5. Statistical Analysis

Data analysis was performed using IBM SPSS Statistics for Windows, version 28 (IBM, Armonk, NY, USA). Categorical data were assessed using chi square and Fisher’s exact tests, while continuous variables were evaluated with the Mann–Whitney U test. A *p*-value of less than 0.05 was considered statistically significant. Logistic regression modeling was applied to determine potential risk factors for pulmonary hemorrhage [36]. Variables that met the inclusion threshold of *p* < 0.1 (or *p* < 0.25) in the univariate analysis were subsequently incorporated into the multivariate regression model for further evaluation.

## 3. Results

### 3.1. Study Population

**Figure 7:**
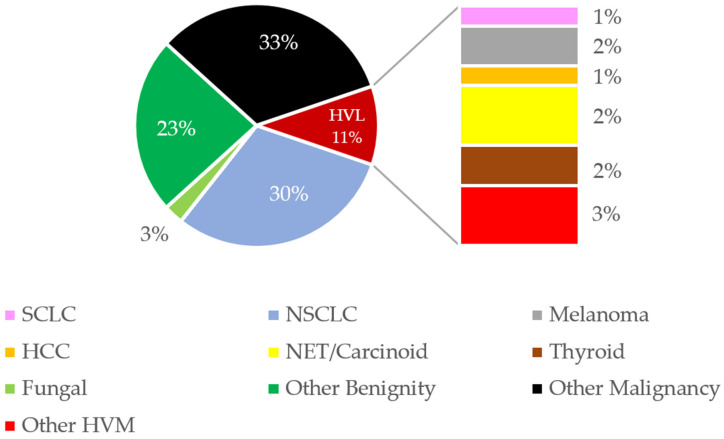
Histological findings of the lung biopsies. HVL = hypervascular lesions; other HVM = hypervascular metastasis like sarcoma; SCLC = small cell lung cancer; NSCLC = non-small cell lung cancer; HCC = hepatocellular carcinoma; NET = neuroendocrine tumor; RCC = renal cell carcinoma. Percentages are rounded. Metastases from breast cancer and RCC are not included in the pie chart as no cases were recorded.

Age and sex did not differ significantly between the groups of patients. Lesion size, lesion location, needle system, needle size, number of samples, biopsy angle, the skin-to-lesion, pleura-to-lesion distance, existing emphysema, and pathological findings were normally distributed in both groups (Table 1). A total of 74% of the biopsied lung nodules were malignant. Most of these were metastases, and about one-third were primary lung tumors. About a quarter of all biopsies were benign lesions (Figure 7).

**Figure 8:**
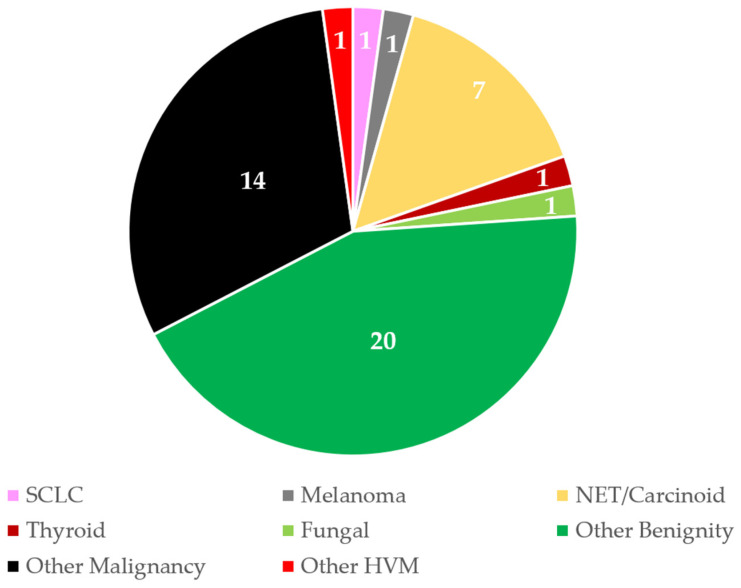
Histological findings of lung biopsies with GGO in the access route. Total number = 46; 40% of all performed lung biopsies. The numbers in the pie chart correspond to the frequency. GGO = ground-glass opacity; other HVM = hypervascular metastasis like sarcoma; SCLC = small cell lung cancer; NSCLC = non-small cell lung cancer; HCC = hepatocellular carcinoma; NET = neuroendocrine tumor; RCC = renal cell carcinoma. No cases were recorded for NSCLC, HCC, RCC, or breast cancer metastases.

A significant proportion (40%) of biopsied lesions had GGO in the access route. A large proportion of these, namely 44%, were benign lesions and almost a third were lesions that were summarized as other malignancies (Figure 8).

### 3.2. Hemorrhage After CT-Guided Lung Biopsy

Pulmonary hemorrhage (Grade 2 or higher) occurred in 26% (30/115) of cases. Five resulted in Grade 3 hemorrhage (4%) and three in Grade 4 hemorrhage (3%). Three patients required hospital admission and monitoring, and one patient underwent thoracoscopic intervention and transfusion. In contrast, 17% (19/115) of patients showed no detectable hemorrhage (Grade 0), while 57% (66/115) exhibited small microhemorrhages along the access route (Grade 1).

The univariate analysis showed a significant association between hemorrhage beyond 2 cm around the needle path and patient positioning in a dependent location, regardless of lesion location in the upper or lower lobe (*p* = 0.034). In addition, hemorrhage occurred more frequently when the distance from the pleura to the lesion was larger (*p* = 0.035). Ground-glass opacities in the access route were significantly associated with pulmonary hemorrhage (*p* = 0.001, Table 1).

### 3.3. Association of Lesion Characteristics and Technical Parameters with the Occurrence of Pulmonary Hemorrhage Grade 2 or Higher

Multivariable logistic regression analysis confirmed that GGOs in the access route before biopsy (OR 5.169, 95% CI 1.889–14.144, *p* = 0.001) and biopsy in dependent lung areas (OR 4.064, 95% CI 1.477–11.186, *p* < 0.001) were associated with pulmonary hemorrhage Grade 2 or higher, independent of lesion size, distance to pleura, upper or lower lobe location, or the type of lesion (Table 2). A model was fitted with an R^2^ = 0.332, *p* = 0.001. Cohen’s f2 is 0.49, corresponding to a strong effect [37].

## 4. Discussion

This study showed for the first time that GGOs in the access route of CT-guided lung biopsy are associated with a 5-fold increased risk of post-interventional pulmonary hemorrhage Grade 2 or higher, while biopsy of target lesions in dependent areas of the lung is associated with a 4-fold increased risk. This finding is highly significant, as the needle tract can be selected to avoid GGOs in the access path, particularly with new laser-guided CT systems that enable double oblique needle access paths. Additionally, patients may be placed in a supine, prone, or lateral position to optimize access to lesions in dependent or non-dependent lung areas. In fact, patient positioning and needle trajectory are more critical than the lobar location or lesion size in minimizing the risk of bleeding. Fortunately, pulmonary hemorrhage Grade 2 is rarely clinically significant. However, Grade 3 and 4 bleeding may occur, and are potentially lethal.

The results of this study support and expand upon the findings of previous research. Zhu et al. found a higher risk of pulmonary hemorrhage in lower-lobe lesions and lung metastases [20], which is consistent with our findings, as metastases tend to be more prevalent in the basal lung regions [38]. However, we found that the risk is not associated with the target lesion’s lobar location, but more whether the target lesion is in a dependent or non-dependent lung area during biopsy. This observation may be attributed to hydrostatic effects, resulting in increased perfusion [39,40,41] and higher pulmonary vessel density in dependent lung areas [42]. In addition, our results suggest that it is the GGO in the access route rather than the exact type of the target lesion that is associated with the risk of pulmonary hemorrhage. This aligns with existing research, which has linked subsolid lesions to increased hemorrhage severity [2] and a risk of severe hemoptysis [12]. The reason for the higher bleeding risk in lung areas with GGOs is most probably vascular fragility [14] and local inflammation with corresponding hyperperfusion in those areas [43]. Compared to previous studies, Grade 2 or higher pulmonary hemorrhage was observed in 26% of cases in our study, whereas other studies reported slightly lower rates ranging from 17.1% to 20.9% [2,9]. Our rigorous standard procedure may explain this difference in acquiring a complete post-interventional thoracic CT scan five minutes after needle withdrawal. Without such a standardized protocol, pulmonary hemorrhage may be underreported, as also previously suggested by Nour et al. [1].

Another key factor is that we frequently positioned the target lesion in a dependent region before biopsy to minimize pneumothorax risk. The risk of pneumothorax is lower when biopsies are performed in the dependent two-thirds of the lung, due to a lower negative pleural pressure. Interventionalists have therefore the difficult choice to decide whether they biopsy in the dependent two-thirds of the lung to avoid pneumothorax, or the non-dependent third of the lung to avoid pulmonary hemorrhage. A possible good solution may be to avoid GGOs in the access path and positioning the target lesion in the middle third of the lung to avoid pneumothorax and bleeding. Whether this approach may reduce the risk of clinically significant pulmonary hemorrhage Grade 3 and 4 compared to biopsy in the lower third of the lung needs to be confirmed in larger, prospective trials. It is also important to consider that such specific positioning techniques may not be suitable for all patients, particularly those who are critically ill and unable to tolerate the lateral position. Additionally, identifying the optimal access route with adapted patient positioning may result in a longer procedure time and higher radiation exposure. Despite the findings of this study, the most appropriate position and access route can only be determined after the planning scan for the biopsy has been completed. Furthermore, trained nursing staff play a crucial role in assisting the interventionalist in efficiently positioning the patient within limited space constraints while ensuring accessibility and procedural feasibility.

Our study has several limitations. First, it was a retrospective analysis conducted at a single center with a relatively small sample size. Second, a simplified model of the gravitational effect in specific patient positions was used, as an invasive perfusion pressure measurement was not feasible. However, such a simple model has the advantage of being easily implemented into the clinical routine. Finally, we recognize that there are additional variables, such as technical differences between different centers and operator skills [44], that are challenging to evaluate and have not been investigated in this study. External validation by a larger, multicenter analysis is therefore needed.

## 5. Conclusions

In conclusion, GGOs in the access route and biopsy in highly perfused dependent lung areas represent two independent risk factors for pulmonary hemorrhage during CT-guided lung biopsy. Careful patient positioning and planning of the access route may mitigate these risks.

## Figures and Tables

**Table 1:** Patient Demographics and Lesion Characteristics. Unless stated otherwise, data are number of biopsies. ± standard deviations. X2 (R X 2), Fisher’s exact test and the Mann–Whitney U test were used to calculate the statistical difference between groups of categorical, dichotomous, and continuous variables, respectively. Data are means ± standard deviations. * Statistically significant (defined as *p* < 0.05); y = year; G = gauge; mm = millimeter; SL = skin to lesion; PL = pleural to lesion, UL = upper lobe; LL = lower lobe; D = dependent; NSCLC = non-small cell lung cancer.

Survey of Lung Biopsies							
Parameter	All (n = 115)		None or Grade 1 Hemorrhage (n = 85)		Grade 2 Hemorrhage or Higher (n = 30)		*p* Value
Sex							
Female	48	42%	35	41%	13	43%	1
Male	67	58%	50	59%	17	57%	
Age (y)	67.23	±12.24	66.67	±13.05	68.83	±9.556	1
Lesion size (mm)	25.03	±18.81	26.8	±20.99	20.03	±8.83	0.18
Needle size							1
18 G	70	61%	52	61%	18	60%	
20 G	45	39%	33	39%	12	40%	
Biopsy system							0.638
side-cut	32	28%	25	29%	7	23%	
full-core	83	72%	60	71%	23	77%	
Number of samples							0.217
1 and 2	22	19%	17	20%	5	17%	
3	55	48%	39	46%	16	53%	
4	24	21%	19	22%	5	17%	
5 and 6	14	12%	10	12%	4	13%	
Biopsy angle (degree)	64.4	±18.10	65.02	±17.65	62.67	±19.54	0.593
Distance SL (mm)	63.11	±21.32	61	21.15	69.1	±21.01	0.074
Distance PL (mm)	16.57	±14.33	15.26	±14.84	20.3	±12.24	0.035 *
Lesion location							0.09
UL	53	46%	35	41%	8	27%	
LL	62	54%	50	59%	12	40%	
Lesion location in D area	53	46%	34	40%	19	63%	0.034 *
Emphysema	22	19%	14	16%	8	27%	0.28
Ground-glass in the access route	46	40%	26	31%	20	67%	0.001 *
Pathological findings							
Hypervascular lesions	12	10%	11	13%	1	3%	0.18
Other malignancy	38	33%	29	34%	9	30%	0.822
NSCLC	34	30%	25	29%	9	30%	1
Adenocarcinoma	33	29%	21	25%	12	40%	0.161
Metastasis	44	38%	37	44%	7	23%	0.079
Benign	30	26%	23	27%	7	23%	0.811

**Table 2:** Results of Multivariate Logistic Regression Analysis for Pulmonary Hemorrhage Grade 2 or Higher. The total number of cases in the cohort for the multivariable analysis was n = 115; B = regression coefficient; S.E. = standard error; df = degree of freedom; CI = confidence interval; D = dependent; LL = lesion location in lower lobe; GG = ground glass; PL = pleural to lesion; * statistically significant (defined as *p* < 0.05).

	B	S.E.	Wald Test	df	*p* Value	Odds Ratio	95% CI	
Variable							−	+
Lesion location in D area	1.402	0.517	7.369	1	0.007 *	4.064	1.477	11.186
LL	−0.98	0.505	3.765	1	0.052	0.375	0.139	1.01
Lesion size	−0.027	0.023	1.63	1	0.244	0.974	0.931	1.018
GG in the access route	1.643	0.514	10.233	1	0.001 *	5.169	1.889	14.144
Distance PL	0.027	0.019	2.012	1	0.156	1.027	0.99	1.066
Metastasis	−0.706	0.543	1.689	1	0.194	0.494	0.17	1.431

## Data Availability

Dataset available on request from the authors.
